# Identification and Classification of Small Sample Desert Grassland Vegetation Communities Based on Dynamic Graph Convolution and UAV Hyperspectral Imagery

**DOI:** 10.3390/s23052856

**Published:** 2023-03-06

**Authors:** Tao Zhang, Yuge Bi, Xiangbing Zhu, Xinchao Gao

**Affiliations:** College of Mechanical and Electrical Engineering, Inner Mongolia Agricultural University, Hohhot 010018, China

**Keywords:** desert steppes, hyperspectral remote sensing, unmanned aerial vehicle, dynamic graph convolution, classification and identification

## Abstract

Desert steppes are the last barrier to protecting the steppe ecosystem. However, existing grassland monitoring methods still mainly use traditional monitoring methods, which have certain limitations in the monitoring process. Additionally, the existing deep learning classification models of desert and grassland still use traditional convolutional neural networks for classification, which cannot adapt to the classification task of irregular ground objects, which limits the classification performance of the model. To address the above problems, this paper uses a UAV hyperspectral remote sensing platform for data acquisition and proposes a spatial neighborhood dynamic graph convolution network (SN_DGCN) for degraded grassland vegetation community classification. The results show that the proposed classification model had the highest classification accuracy compared to the seven classification models of MLP, 1DCNN, 2DCNN, 3DCNN, Resnet18, Densenet121, and SN_GCN; its OA, AA, and kappa were 97.13%, 96.50%, and 96.05% in the case of only 10 samples per class of features, respectively; The classification performance was stable under different numbers of training samples, had better generalization ability in the classification task of small samples, and was more effective for the classification task of irregular features. Meanwhile, the latest desert grassland classification models were also compared, which fully demonstrated the superior classification performance of the proposed model in this paper. The proposed model provides a new method for the classification of vegetation communities in desert grasslands, which is helpful for the management and restoration of desert steppes.

## 1. Introduction

Desert steppes are the last barrier to the degradation of grasslands into deserts, and effective monitoring of their grassland vegetation community structure is an effective means to prevent and manage grassland degradation [1]. In recent years, the combined effects of global climate change and human factors have intensified the process of grassland degradation [2]. Grassland degradation not only reduces grassland productivity, but also decreases biodiversity, which is a serious threat to grassland ecosystems [3]. Therefore, it is particularly important to explore an efficient and high-precision method to monitor grassland vegetation communities.

At the present stage, grassland monitoring is carried out mainly by arranging fixed stations. This monitoring method has high accuracy and reliable data, but it also has many limitations, such as long monitoring intervals, high labor costs, and labor-intensive and time-consuming problems [4]. Satellite remote sensing is also commonly used in grassland monitoring studies, but, due to its spatial resolution limitation, it is limited to grassland cover monitoring studies, while it cannot meet the requirements for monitoring fine vegetation communities [5]. An unmanned aerial vehicle (UAV) hyperspectral remote sensing platform with high spatial resolution, rich spectral information, wide monitoring range, and flexible operation is the first choice to replace traditional ground-based grassland vegetation community monitoring means [6,7,8].

The deep learning method is a powerful feature extractor, is good at handling complex multidimensional data structures, and is one of the effective methods for processing hyperspectral imagery (HSI) data [9,10]. Currently, convolutional neural networks (CNN) are widely used in processing hyperspectral data [11], and the spectral features and spatial texture features of hyperspectral image data can be effectively extracted for classification by a certain number of convolutional kernels. For example, Jia et al. [12] used CNN to extract spectral features and achieved better performance than SVM. However, ordinary CNN only convolves regular square regions, which cannot adaptively capture the geometric variations of different target regions in hyperspectral images, and they are prone to incorrect classification. Graph convolutional networks (GCNs), which can effectively aggregate feature information of itself and neighboring graph nodes, are used mainly on graph structure classification tasks [13,14,15], and are less often applied to the classification of hyperspectral images. In recent years, some scholars have started applying GCNs to hyperspectral image classification; for example, Xibing Zuo et al. [16] achieved better classification performance than CNN under small samples by constructing hyperspectral data graph structures and using a GCN to classify them. However, existing hyperspectral data composition methods need to calculate the distance relationship between global pixel points for composition, which will substantially increase the memory and time required for the analysis, and this is not conducive to model training and mobile deployment. Furthermore, the constructed graph structure is a fixed graph, which cannot adaptively fuse the information on the features of different nodes and easily introduces some erroneous nodes for aggregation, thus causing degradation of the classification performance of the model.

To address the above problems, this paper uses a UAV hyperspectral remote sensing platform for the collection of data from the desert steppe vegetation community and proposes a spatial neighborhood dynamic graph convolution network (SN_DGCN) classification model for the classification task of vegetation communities. The network constructs spatial neighborhood graphs by sliding windows of fixed sizes for them, while GCN is used to aggregate node information. In addition, different graph structures are constructed for different layers in the GCN to be adaptive to the relationship between different pixel points. The purpose of this paper is to discuss the application of graph convolution in the classification task of UAV hyperspectral remote sensing data of grassland vegetation communities in order to provide a new technical means for the efficient and high-precision dynamic monitoring of desert steppes. The contributions of this study are as follows:

1. To provide a new high-efficiency and high-precision monitoring method for grassland degradation monitoring. The limitations of manual survey and satellite remote sensing are addressed.
2. Propose a spatial neighborhood-based dynamic graph convolution classification algorithm. The classification accuracy of the model is greatly improved under small samples, which is better than other related deep learning classification methods.
3. A method for constructing graph structures in the spatial neighborhood is proposed. The method effectively alleviates the large memory consumption and time cost of graph convolutional networks in hyperspectral images.

## 2. Materials and Methods

### 2.1. Overview of the Study Area

The present study area is in the Gegenthala grassland in central Inner Mongolia, China, with the geographical coordinates of 111°52′47″ E and 41°46′48″ N. The area has long winters and short summers, the average annual temperature is at 3.5° C, the annual precipitation is about 260 mm, the elevation is about 1456 m, and it belongs to the middle temperate continental climate. The type of grassland in the study area belongs to the desert steppe of *Stipa breviflora* [17], the surface vegetation is sparse and low, and the average height of the vegetation is approximately 8 cm. The vegetation community in the study area can be divided into constructive species, dominant species, and companion species.

The constructive species are the dominant species in the dominant layer of vegetation communities and can determine the community structure and internal environmental conditions [18]. The dominant species have a high abundance in vegetation communities and have significant control over the formation of vegetation community structure and vegetation community environment [19]. The companion species, also known as accessory species, often exist alongside the dominant species and have no major influence on the processes and functions of the community. According to the survey, the constructive species in the study area were *Stipa breviflora*, the dominant species were *Cleistogenes songorica* and *Artemisia frigida*, and the companion species were *Neopallasia pectinata*, *Leymus chinensis*, and *Ceratoides latens*.

### 2.2. Experiment Equipment

**Figure 1:**
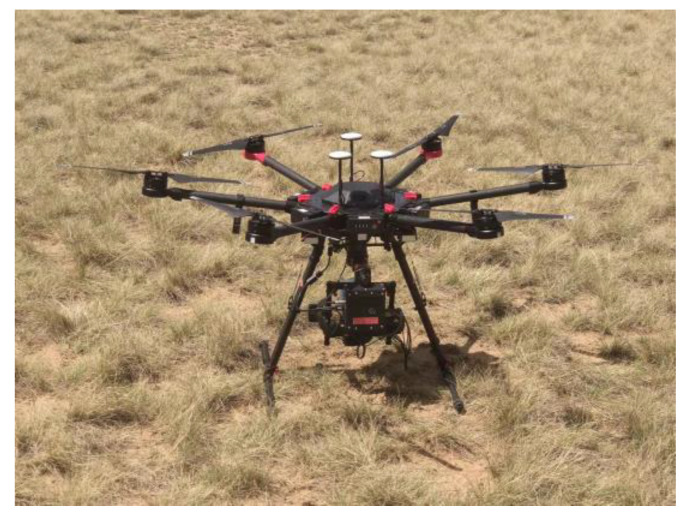
UAV hyperspectral remote sensing system.

The instruments used in this study were mainly a hyper-spectrometer, a UAV, and a gimbal, as shown in Figure 1. Among them, the hyper-spectrometer is a Gaiasky-mini hyper-spectrometer produced by Dualix Spectral Imaging. The spectral range of this spectrometer is between 400 and 1000 nm, with 1392 spatial dimensional pixels, a maximum of 1040 bands, and a single image acquisition time of about 6 s. The UAV is a DJI M600 Pro six-rotor UAV with a professional A3pro flight control system, which can achieve autonomous flight routes. The maximum load is 6 kg, and it can fly continuously for 16 min at full load. The gimbal is a DJI Ronin-M-type gimbal.

### 2.3. Data Collection

Based on the vegetation growth cycle and climatic factors in the study area, field data collection was conducted in July 2021. The collection should ensure sufficient sunlight and avoid interference from shadows, so the collection time was set between 10:00 to 14:00 Beijing time, China. In addition, the collection conditions should be satisfied with cloudless and clear weather, wind speeds less than level 3, and natural light. In the 2.5 hm^2^ sample area, a total of 40 1 m × 1 m quadrats were placed, including 30 quadrats of dominant species, constructive species, and companion species, and 10 mixed quadrats. At the same time, ground mats were used to mark the samples, GPS was used to record the geographic coordinates of the center of the samples, and the vegetation types and coverage in the samples were recorded.

After several tests, a single hyperspectral image size of 696 lines × 775 samples × 256 bands was established while ensuring acquisition efficiency and accuracy; the UAV flight altitude was set at 30 m and the spatial resolution was 2.3 cm. Reflectance correction was required before and after each flight. Each sample was acquired at least three times.

### 2.4. Data Processing

**Figure 2:**
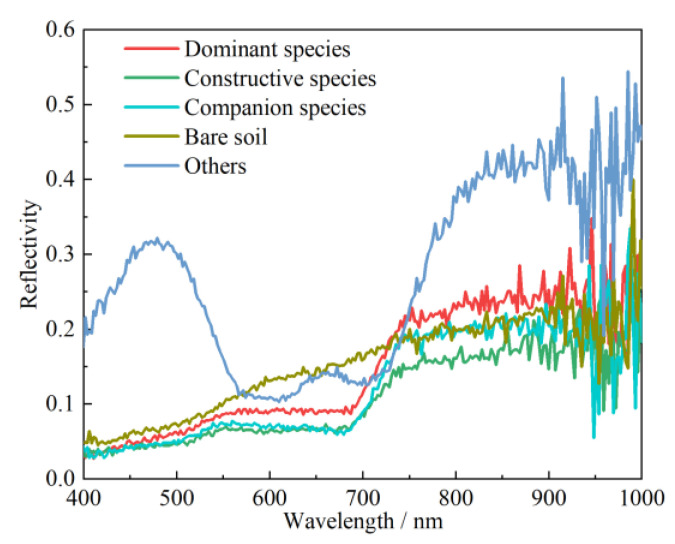
Spectral curve after reflectivity correction.

The acquired hyperspectral data needed to be manually checked to reject poorly imaged data. The remaining data were reflectance corrected by the Spec-VIEW software. The spectral curves of each type of feature after reflectance correction are shown in Figure 2. As can be seen in Figure 2, the reflectance values of 51 bands after the wavelength 886.6 nm show a wide range of fluctuations, which were mainly caused by a large amount of noise. Therefore, to ensure the accuracy of the experimental results, these bands containing a large amount of noise should be removed. Finally, 205 bands were retained for the subsequent experimental analysis.

Based on the extensive ground survey, samples were marked by ArcGIS 10.7 and ENVI 5.3 software. Finally, a total of 3459 samples were obtained, including 1365 dominant species, 811 constructive species, 755 companion species, 284 bare soil, and 244 other samples (ground mats, litter, etc.).

As can be seen in Figure 2, the spectral curves of the other features have greater differences than those of the other four features, which have higher reflectance values. The spectral curves of the bare soil do not have large fluctuations and increase slowly in a straight line. In addition, the spectral curves of bare soil and vegetation were significantly different between the wavelengths of 545.8~740 nm. For vegetation communities, the wavelengths were more similar between vegetation communities between wavelengths of 400~545.8 nm. After 545.8 nm, the reflectance values of the dominant species were significantly higher than those of the constructive and companion species. After 740 nm, the reflectance values of the companion species were higher than those of the constructive species, but lower than those of the dominant species.

## 3. Principle of the Algorithm

### 3.1. Construction of Spatial Neighborhood Graph

In hyperspectral data, the pixel points are sampled point-by-point in their spatial dimension through a sliding window of H × W size. The sampled data are called the local blocks, which can be represented by a three-dimensional matrix $X\in {\mathbb{R}}^{H\times W\times C}$, where *H* × *W* is the spatial size, the height and width of the data, respectively, and *C* is the spectral dimension. Here, *H* = *W* is an odd number and is the size of the sliding window. For the convenience of representation, the size of the sliding window is represented by w in the subsequent analysis.

The dimensions of the three-dimensional matrix X are converted into a two-dimensional matrix ${Χ}^{\prime }\in {\mathbb{R}}^{C\times n}$, where $n=H\times W=w^{2}$ is the number of pixels in the neighborhood of that space. Thus, the features of a point within a sliding window can be represented by $P=\left\{x_{1}^{\prime },x_{2}^{\prime },\ldots ,x_{i}^{\prime }\right\}\in {\mathbb{R}}^{C},i=1,2,\ldots ,n$, and *C* is the feature dimension of the point, as well as the spectral dimension of the point. In a convolutional neural network, each layer of input operates on the output of the previous layer, so usually *C* also denotes the feature dimension of a given layer.

**Figure 3:**
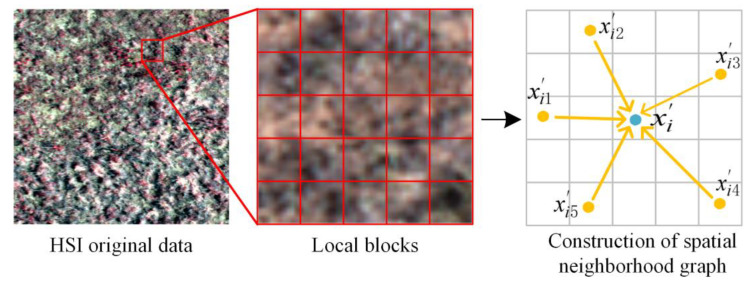
Construction of spatial neighborhood graph.

In the construction of the spatial neighborhood graph, the Euclidean distance is used as the distance metric function to calculate the distance relationship between the pixel points in ${Χ}^{\prime }$. Meanwhile, the k nearest neighbor nodes $x_{i}^{\prime },\;j=1,\;2,\;\ldots ,\;k$ of the pixel point $x_{i}^{\prime }$ are taken for directed edge composition, and Figure 3 shows an example of the construction of the spatial neighborhood graph. A directed graph G of spatial neighborhood hyperspectral data can be represented by $G=\left(V,\;E\right)$, where $V=\left\{x_{1}^{\prime },x_{2}^{\prime },\ldots ,x_{i}^{\prime }\right\}$ and E∈V×V denote the set of nodes and the set of edges, respectively. In the simplest case, after the construction of the spatial neighborhood graph, there is an edge set $\left(x_{i}^{\prime },x_{i1}^{\prime }\right),\;\ldots ,\left(x_{i}^{\prime },x_{ik}^{\prime }\right)$ that can be represented so that its $x_{i1}^{\prime },\ldots ,x_{ik}^{\prime }$ is closest to $x_{i}^{\prime }$.

### 3.2. Edge Convolution Block

In graph convolution, for the extraction of edge features, in addition to the features of nodes and neighboring nodes, the features of the nodes themselves should be included [20]. Therefore, the extraction function of the edge features is defined as follows:

(1) $$e_{ij}=h_{\Theta }\left(x_{i}^{\prime },x_{j}^{\prime }-x_{i}^{\prime }\right),$$

where $h_{\Theta }:\;{\mathbb{R}}^{C}\times {\mathbb{R}}^{C}\to {\mathbb{R}}^{c^{\prime }}$ is a set of nonlinear functions with learnable parameters $\Theta =\left(\theta_{i},\ldots ,\theta_{k}\right)$, and $e_{ij}\in {\mathbb{R}}^{C\times n\times k}$ is the extracted edge feature. Here, $x_{i}^{\prime }$ is used to capture the global information of the graph and $x_{j}^{\prime }-x_{i}^{\prime }$ is used to capture the local information of the graph.

Here, 2D_Conv+BN+ReLU is chosen to represent $h_{\Theta }$, where 2D_Conv is a 1 × 1@□ 2D convolutional kernel, □ represents the number of convolutional kernels, BN is the batch normalization function, and ReLU is the activation function. Then, Equation (1) can be expressed by the following equation:

(2) $$e_{ij}=ReLU\left(BN\left(2D\_Conv\left(x_{i}^{\prime },x_{j}^{\prime }-x_{i}^{\prime }\right)\right)\right),$$

After the edge features are extracted, they also need to be aggregated with the edge features in the channel dimension. Here, the maximum pooling function is chosen to aggregate the main features of the edges and, thus, represent the features of the subgraph. This operation not only reduces the redundant information of the data, but also has displacement invariance (the output of the result will not be affected by the different order of the nodes). The output of features after maximum pooling is expressed as $e_{ij}^{\prime }\in {\mathbb{R}}^{C\times n}$ with the following equation:

(3) $$e_{ij}^{\prime }=\underset{j:\;\left(i,\;j\right)\in E}{\max}e_{ij},$$

**Figure 4:**
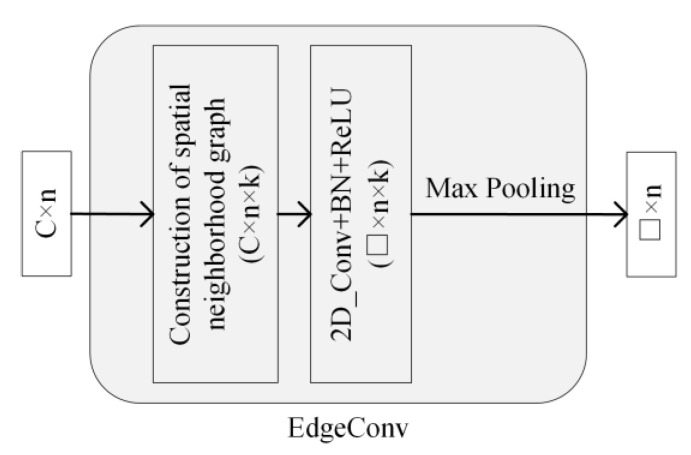
Edge convolution block.

The above process from the construction of the spatial neighborhood graph to the aggregation of edge features is called edge convolution (EdgeConv). An EdgeConv block can be represented by Figure 4, where □ represents the number of 2D convolution kernels.

### 3.3. Spatial Neighborhood Dynamic Graph Convolution

**Figure 5:**
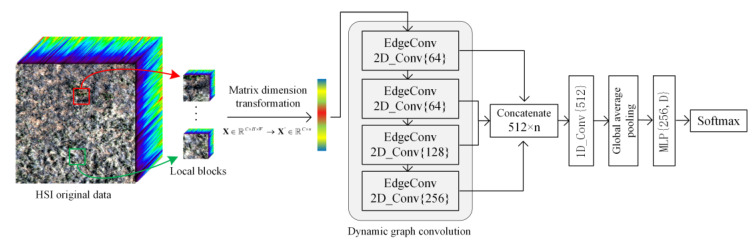
SN_DGCN classification network structure.

The SN_DGCN classification model proposed in this paper contains four layers of EdgeConv blocks, and each layer produces different graphs. The number of 2D convolution kernels is set to 64, 64, 128, and 256 in the four layers of EdgeConv blocks, respectively. After constructing four layers of EdgeConv blocks, the feature graphs output from each layer is concatenated into channel dimensions. At the same time, the connected data are passed into a 1D convolutional layer with a convolutional kernel size of 1 for feature fusion across channels, and the feature map dimension obtained after fusion is $512\times w^{2}$. The obtained feature map is then globally fused by a global average pooling layer for all pixel points to obtain a feature vector of dimension 512. Finally, the final classification is performed by passing into a multi-layer perceptron (MLP), where the number of neurons per layer is defined as {256, D}, where D = 5 represents the number of predicted categories. Also, the dropout layer is introduced in the MLP to alleviate the overfitting problem of the network. The dropout here is a regularization method that refers to the random removal of neurons with a certain probability in deep learning training, which helps to improve the model generalization ability. In addition, BN and ReLU layers are added to the perceptual and 1D convolutional layers, except for the last perceptual layer of the MLP. Finally, the output of the last layer of the MLP is passed into the Softmax function for classification. The proposed SN_DGCN classification network structure is shown in Figure 5, in which 2D_Conv{64} and 1D_Conv{512} represent 2D convolutional kernels of size 1 × 1 and 1D convolutional kernels of size 1, respectively.

## 4. Experimental Results and Analysis

The experimental software was the Pytorch framework for model construction, and the programming language was Python. The computer hardware configuration was i7-11800H CPU, GeForce RTX 3060 GPU, and 16 GB of memory. The learning rate of model training was 0.001, the number of iterations was 200, the Adam algorithm was selected to optimize the loss, the cross-entropy loss function was selected to calculate the model prediction loss, and the batch size was 128. In this paper, three metrics, overall accuracy (OA), average accuracy (AA), and Kappa coefficient, were selected to evaluate the classification performance of the model. In addition, the sliding window size w and the number of nearest neighbor nodes k were initially set to 7 and 10, respectively. In the model training process, 10 samples of each type of ground objects were randomly selected for training, and the rest of the samples were used for testing (as shown in Table 1). Meanwhile, the accuracy of the test set was recorded at every five iterations, and the model with the highest accuracy in the test set was selected as the final model for this training. To ensure the reliability of the experimental data, each experiment was repeated five times, and the average value was taken as the final result.

### 4.1. Analysis of Experimental Parameters

#### 4.1.1. Analysis of the Numbers of Neighboring Nodes *k*

**Figure 6:**
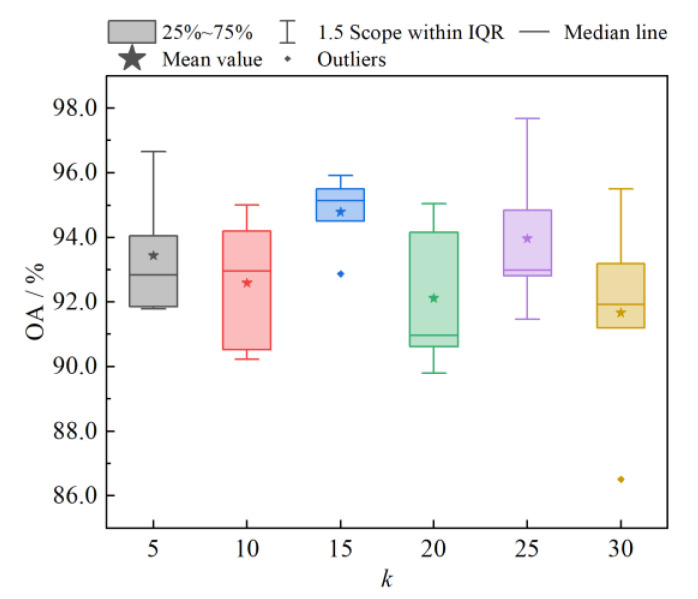
Box plots of OA with different numbers of neighborhood nodes *k*.

In the construction of the spatial neighborhood graph, the number of neighborhood nodes *k* has an important influence on the performance of the model classification. If the value of *k* is too large, it will bring in too many redundant nodes for aggregation, which will cause the degradation of the classification performance; if the value of *k* is too small, it will lose some node information and cannot fully express the dependency relationship among the pixels, which is not conducive to the improvement of the model classification performance. Figure 6 shows the OA box plots for different numbers of neighborhood nodes *k*. From the figure, it can be seen that when *k* = 15, the model had the highest average OA and the classification performance was more stable. Therefore, the number of neighborhood nodes was chosen to be 15 in the subsequent analysis experiments.

#### 4.1.2. Analysis of the Sliding Windows *w*

**Figure 7:**
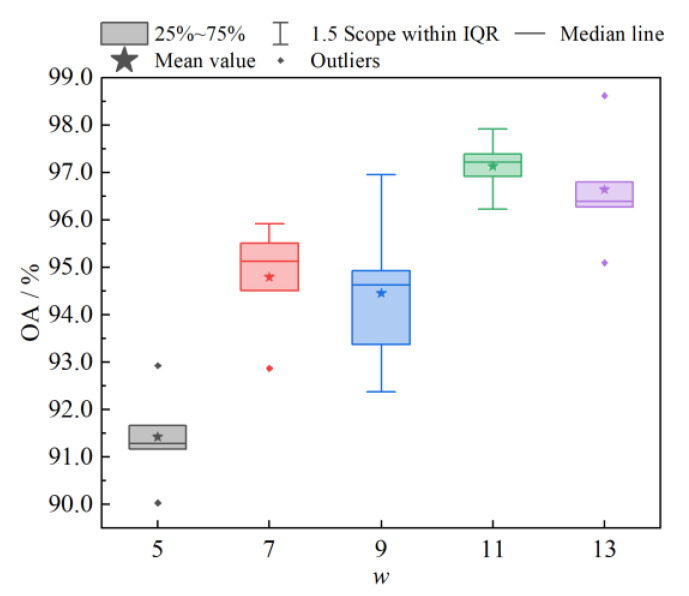
Box plots of OA with different sliding windows *w*.

The larger the sliding window *w* is, the larger the perceptual field of view will be, and, at the same time, it becomes easier to capture distant neighbor nodes to help its classification in the composition process. However, a too large *w* will decrease the classification performance of the network model and also introduce redundant information, as well as increase the computational cost of the model. Figure 7 shows the OA box plots for different *w* values. From the figure, it can be seen that too large or too small *w* values were not conducive to the stability of the classification performance of the model and were prone to outliers. When *w* = 11, the average OA of the model was the highest and the classification performance was more stable. Therefore, the sliding window size was chosen to be 11.

### 4.2. Model Classification Performances Compare Experiments

To verify the effectiveness of the proposed method (SN_DGCN) in this paper, a total of six classification models, MLP [21], 1DCNN [22], 2DCNN [23], 3DCNN [24], Resnet18 [25], and Densenet121 [26], were selected for comparison experiments. The parameters of the selected comparison models were all the same as in the original paper. In addition, to evaluate the effectiveness of dynamic graph convolution in the proposed SN_DGCN network model, a set of SN_GCN network comparison experiments without dynamic graphs was added. The means and standard deviations of the classification accuracy of all models are shown in Table 2, all experimental results are expressed as percentages, and the model with the best classification effect is shown in bold.

It can be seen from Table 2 that the MLP and 1DCNN had the worst classification performance in the eight groups of comparison experiments. The OA, AA, and Kappa of the MLP were 69.16%, 71.03%, and 57.77%, respectively. The OA, AA, and Kappa of the 1DCNN were 68.24%, 72.42%, and 58.88%, respectively. Compared to the 2DCNN classification model, the OA, AA, and Kappa of the 3DCNN improved by 10.3%, 11.16% and 14.26%, respectively, over the 2DCNN, which indicated that the convolutional kernel with cubic structure was more suitable for the classification of hyperspectral images. The overall classification performance of the Resnet18 classification model was higher than that of the four MLP, 1DCNN, 2DCNN, and 3DCNN classification models, thus indicating that the residual structure had a better effect on feature extraction and could effectively alleviate the overfitting problem in the network. The Densenet121 classification model improved the transfer between features due to dense connectivity, and its OA, AA, and Kappa improved by 4.55%, 1.6%, and 5.83%, respectively, over the Resnet18 classification model. The overall classification performance of the SN_GCN classification model without a dynamic graph was much higher than the six classification models of MLP, 1DCNN, 2DCNN, 3DCNN, Resnet18, and Densenet121, whose OA, AA, and Kappa were 95.9%, 95.95%, and 94.37%, respectively, which indicated that the graph convolution could make full use of the dependency of neighborhood node relationships for classification and could effectively aggregate the features of neighboring nodes to improve the classification performance of the models. The classification effect of the proposed SN_DGCN classification model performed the best among all the models compared, and its OA, AA, and Kappa were 97.13%, 96.50%, and 96.05%, respectively. Compared with the SN_GCN classification model, the OA, AA, and Kappa of the SN_DGCN were improved compared to the SN_GCN by 1.23%, 0.55%, and 1.68%, thus indicating that the aggregation of the features of neighboring nodes using a dynamic graph was effective and could accurately extract the feature relationships between neighboring pixel points. Meanwhile, it can be seen from all the comparison experiments that the graph convolutional network classification performance had a huge improvement over the ordinary convolutional network, which shows that the aggregation by extracting the neighborhood node features could effectively eliminate the redundant background information and make it easier to extract the features of irregular ground objects, such as desert steppes.

### 4.3. Model Comparison Experiment with Different Training Samples

**Figure 8:**
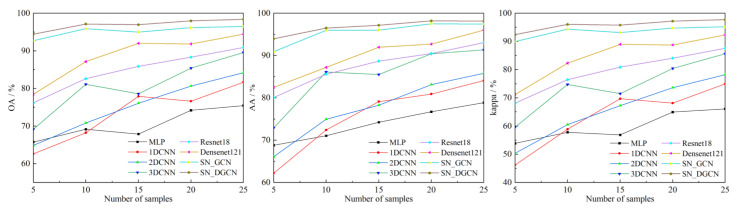
Classification accuracy of different training samples.

Different numbers of training samples have an important impact on the classification performance of the model. Generally speaking, too few training samples may lead to poor generalization ability of the model, and too many training samples can effectively improve the classification performance of the model. However, in practical applications, it is difficult to obtain a large number of training samples for hyperspectral image data because of the difficulty of sample annotation. To further explore the classification performance of the proposed classification model in the case of small samples, five sets of experiments with different sample sizes of 5, 10, 15, 20, and 25 samples per class of ground objects were randomly selected for each class of ground objects. Meanwhile, the same models as in the Section 4.2 were selected for comparison, and the average classification accuracies of the three evaluation indexes of OA, AA, and the Kappa coefficient for the experimental results are shown in Figure 8.

As can be seen in Figure 8, the classification performance of different classification models showed an overall increasing trend as the number of training samples increases, thus indicating that too many training samples can extract more representative information and help improve the classification performance of the model; after the training samples reached 15 for each class of ground objects, the increasing trend started to become flat. The overall classification performance of the three classification models, MLP, 1DCNN, and 2DCNN, did not differ much in the case of too few samples; with the gradual increase in the number of training samples, the difference in the classification performance of the three classification models gradually increased; overall, the classification performance from high to low was 2DCNN, 1DCNN, and MLP. Among the three classification models, 3DCNN, Resnet18, and Densenet121, the classification performance of 3DCNN and Resnet18 was more similar in most cases, and the classification performance gap between Resnet18 and Densenet121 changed steadily; when the number of training samples per class of ground objects was 15, the classification performance gap between the three classification models was the largest; when the number of training samples per class of ground objects was 25, the OA, AA, and Kappa of the Densenet121 exceeded 90%. In both the SN_GCN and SN_DGCN classification models, the OA, AA, and Kappa of different training sample numbers were above 90% and surpassed the classification performance of the remaining comparison models, and the classification performance gap between the two classification models changed smoothly, in which the classification performance of SN_DGCN was better than that of the SN_GCN classification model, thus indicating the effectiveness of the dynamic graph in improving the classification performance of the models; the classification performance of the two classification models tended to be stable after the training sample number of each class of ground objects reached 10. For the SN_DGCN classification model, when the number of samples per class of ground objects was 5, its OA, AA, and Kappa were 94.46%, 93.99%, and 92.44%, respectively; when the number of samples per class of ground objects was 25, its OA, AA, and Kappa were 98.38%, 98.13%, and 97.77%, respectively. Compared to when the number of samples per class of ground objects was 5, the OA, AA, and Kappa improved by 3.92%, 4.14%, and 5.33%, respectively. The results show that the SN_DGCN classification model for different training sample conditions had higher classification accuracy than all the comparison models, and had better generalization ability for the classification of small samples, which is beneficial for the classification task of irregular shapes like desert steppes.

### 4.4. Feature Visualization Analysis

**Figure 9:**
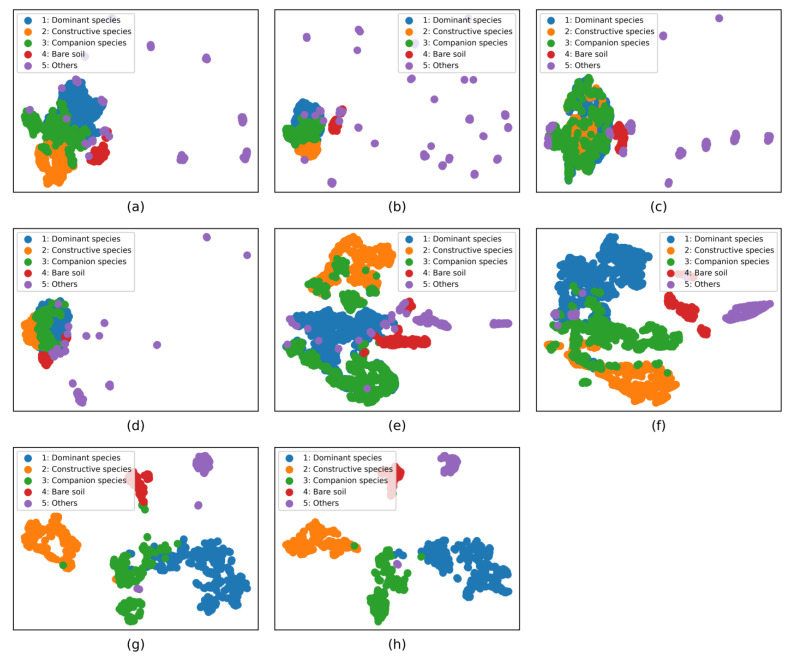
Results of feature visualization for different models. (**a**) MLP, (**b**) 1DCNN, (**c**) 2DCNN, (**d**) 3DCNN, (**e**) Resnet18, (**f**) Densenet121, (**g**) SN_GCN, and (**h**) SN_DGCN.

To effectively analyze the model’s ability to extract features for desert grassland ground objects, the output of the upper layer of the model classification layer was selected for t-SNE [27] visualization, as is shown in Figure 9. From the figure, it can be seen that the four models (MLP, 1DCNN, 2DCNN and 3DCNN) could not effectively distinguish the features of different vegetation communities. In addition, the feature extraction for other ground object samples was more scattered and could not learn effective information. The Resnet18 and Densenet121 models improved the feature learning for vegetation communities compared with the previous four models. However, the two models had too much misclassification for the identification of different vegetation communities. The SN_GCN model learned more focused vegetation community features and could effectively identify different vegetation communities, which indicates that the graph convolution is beneficial for the identification of desert grassland vegetation communities. However, the SN_GCN model was weak in distinguishing dominant species from companion species, and there was partial misidentification between two communities. The feature extraction ability of the proposed SN_DGCN model was better than all the comparison models, and the differentiation ability of the SN_DGCN model for dominant and companion species was significantly improved compared with the SN_GCN model, which indicates that the dynamic graph construction is helpful to improve the classification performance of the model and enhance the model to learn the difference between the features of different vegetation communities.

### 4.5. Discussion

It is the basis of intelligent monitoring of grassland degradation to explore an efficient and high-precision model for identifying desert grassland vegetation communities. In recent years, more and more scholars have started to study the intelligent monitoring methods of grassland degradation. Deep learning has powerful feature extraction ability, and it is effective for improving the recognition accuracy among vegetation by combining deep learning and hyperspectral images. However, due to the irregular distribution of desert grassland vegetation, the vegetation leaves are too narrow and there is strong spectral similarity, which causes great difficulties in recognition and classification among vegetation. Deep learning has good recognition effects compared with traditional image processing methods and machine learning, but it often requires a large number of labeled samples to produce the desired classification effect. However, labeled samples of hyperspectral images are difficult, especially for grasslands, which are more challenging to label.

To reduce the number of markers in samples and enhance the robustness of deep learning models, a SN_DGCN deep learning model was constructed in this paper for the recognition and classification study among vegetation communities in desert grasslands. Through extensive model comparison experiments, we found that the proposed model had better robustness and generalization ability in small samples. The main reason is that the model fully learns the spatial features and spectral features of hyperspectral images. In addition, the neighborhood graph construction approach further strengthens the correlation between pixels and makes full use of the features of neighboring pixels to assist in recognition and classification.

To further illustrate the recognition ability of the proposed model for desert grassland features, the latest deep learning models on grassland feature recognition were selected for comparison experiments, including DGC-3D-CNN [28], DIS-O [29], LGFEN [30], and TAN [31]. It should be noted that the sizes of the sliding windows *w* of the selected models were 7, 5, 15 and 9, respectively, and the experimental results are shown in Table 3. From the table, it can be seen that the proposed SN_DGCN model had the best classification performance, which further proves the ability of the SN_DGCN model to identify desert grassland vegetation communities.

Although the SN_DGCN model proposed in this paper performed well in the desert grassland vegetation classification task, the data used were single month data, thus limiting the monitoring time. In the future, we will consider data collection for different growth stages according to different growth cycles of vegetation to enhance the applicability of the model for multi-time monitoring.

### 4.6. Visualization of Experimental Sample Area Classification

**Figure 10:**
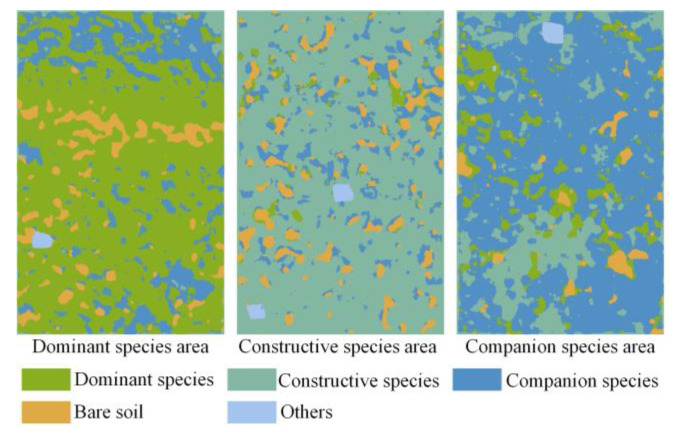
Classification visualization results.

In order to better evaluate the classification effect of the SN_DGCN classification model in practical application, three community sample areas of dominant species, constructive species, and companion species were visualized for classification, as are shown in Figure 10. After comparing with the ground survey records, the spatial distribution between each ground object matched the actual distribution, which shows that the algorithm in this paper has good generalization ability and is advantageous in the task of the classification of vegetation communities in desert steppes.

## 5. Conclusions

A SN_DGCN classification model for desert steppe vegetation communities was proposed for the problems of poor classification accuracy and difficulties in marking hyperspectral image data in the existing desert steppe vegetation communities, and its main model parameters were discussed. Meanwhile, the SN_DGCN classification model was compared with seven different classification models. The results showed that the SN_DGCN classification model had the best classification performance among all the compared models, with OA, AA, and Kappa values of up to 97.13%, 96.50%, and 96.05%, respectively, when only 10 samples of each class of ground objects were available. Furthermore, the classification performance of different classification models was compared for different numbers of training samples; the SN_DGCN classification model had the highest classification accuracy and was more stable for different training samples. This shows that the SN_DGCN classification model is effective in facing the classification of desert steppes and has advantages in the classification task of irregular ground objects. The proposed classification model not only provides a new method for high accuracy classification of desert steppe vegetation communities, but also provides a reference value for other classification tasks of hyperspectral images.

## Figures and Tables

**Table 1:** Number of training set and test set samples.

No.	Class	Training	Test
1	Dominant species	10	1355
2	Constructive species	10	801
3	Companion species	10	745
4	Bare soil	10	274
5	Others	10	234
Total		50	3409

**Table 2:** Comparison of classification performance of different models (%). Note: Bold represents the highest classification accuracy.

No.	MLP	1DCNN	2DCNN	3DCNN	Resnet18	Densenet121	SN_GCN	SN_DGCN
1	70.76 ± 0.09	70.76 ± 0.10	69.98 ± 0.08	72.96 ± 0.06	75.69 ± 0.04	90.20 ± 0.04	96.25 ± 0.02	**98.23** ± 0.01
2	77.95 ± 0.10	71.61 ± 0.12	71.89 ± 0.06	95.31 ± 0.02	96.18 ± 0.03	90.94 ± 0.06	**98.18** ± 0.01	98.15 ± 0.01
3	49.18 ± 0.06	46.98 ± 0.10	59.95 ± 0.07	70.71 ± 0.03	74.66 ± 0.11	75.60 ± 0.05	92.16 ± 0.01	**94.82** ± 0.01
4	100.00 ± 0.00	99.49 ± 0.01	99.27 ± 0.01	97.81 ± 0.03	97.81 ± 0.02	94.60 ± 0.08	**100.00** ± 0.00	**100.00** ± 0.00
5	57.26 ± 0.07	73.25 ± 0.07	73.67 ± 0.09	**93.76** ± 0.03	83.59 ± 0.06	84.62 ± 0.08	93.16 ± 0.07	91.37 ± 0.06
OA	69.16 ± 0.02	68.24 ± 0.03	70.84 ± 0.03	81.14 ± 0.03	82.60 ± 0.02	87.15 ± 0.02	95.90 ± 0.01	**97.13** ± 0.01
AA	71.03 ± 0.02	72.42 ± 0.01	74.95 ± 0.02	86.11 ± 0.02	85.59 ± 0.02	87.19 ± 0.03	95.95 ± 0.01	**96.50** ± 0.01
Kappa	57.77 ± 0.02	58.88 ± 0.04	60.51 ± 0.04	74.77 ± 0.04	76.46 ± 0.03	82.29 ± 0.03	94.37 ± 0.01	**96.05** ± 0.01

**Table 3:** Performance comparison with existing desert grassland deep learning models (%). Note: Bold represents the highest classification accuracy.

No.	DGC-3D-CNN	DIS-O	LGFEN	TAN	SN_DGCN
1	73.22 ± 0.09	81.16 ± 0.07	93.83 ± 0.04	96.37 ± 0.02	**98.23** ± 0.01
2	90.43 ± 0.04	82.82 ± 0.05	**98.30** ± 0.02	98.60 ± 0.01	98.15 ± 0.01
3	56.05 ± 0.09	62.25 ± 0.09	88.08 ± 0.06	90.33 ± 0.05	**94.82** ± 0.01
4	94.38 ± 0.08	100.00 ± 0.09	89.41 ± 0.09	97.59 ± 0.05	**100.00** ± 0.00
5	82.91 ± 0.09	75.30 ± 0.05	83.76 ± 0.07	86.41 ± 0.10	**91.37** ± 0.06
OA	75.88 ± 0.04	78.53 ± 0.02	92.57 ± 0.03	94.99 ± 0.01	**97.13** ± 0.01
AA	79.40 ± 0.03	80.31 ± 0.01	90.68 ± 0.03	93.86 ± 0.02	**96.50** ± 0.01
Kappa	67.41 ± 0.05	70.73 ± 0.03	89.79 ± 0.04	93.09 ± 0.01	**96.05** ± 0.01

## Data Availability

Not applicable.
